# Predicting Work-Life Conflict: Types and Levels of Enacted and Preferred Work-Nonwork Boundary (In)Congruence and Perceived Boundary Control

**DOI:** 10.3389/fpsyg.2021.772537

**Published:** 2021-11-15

**Authors:** Christin Mellner, Pascale Peters, Maria Johanna Dragt, Susanna Toivanen

**Affiliations:** ^1^Department of Psychology, Stockholm University, Stockholm, Sweden; ^2^Strategic Human Resource Management, Nyenrode Business University, Utrecht, Netherlands; ^3^Division of Sociology, Mälardalen University College, Västerås, Sweden

**Keywords:** boundary crossing, boundary management strategies, boundaryless work, inter-domain transitions, work-life balance

## Abstract

In 2020, everyday life changed dramatically for employees worldwide as a result of the outbreak of the Covid-19 pandemic, where an estimated 558 million employees started working from home. The pandemic, therefore, marks a fundamental shift of individuals’ work-nonwork boundaries, which can impact work-life conflict. In particular, the interplay between individuals’ enacted boundaries (degree to which they separate/segment or blend/integrate work-nonwork), preferred boundaries (degree of preferred segmentation or integration of work-nonwork), and perceived control over work-nonwork boundaries, may relate to work-life conflict. This study, the first to the best of our knowledge, examines whether different types and levels of work-nonwork boundary (in)congruence matter for work-life conflict, and whether perceived boundary control moderates these relationships. Boundary (in)congruence represents the degree of (mis)fit between enacted and preferred segmentation or integration. Several types of (in)congruence are distinguished: “segmentation congruence” (enacting and preferring segmentation); “integration congruence” (enacting and preferring integration); “intrusion” (enacting integration but preferring segmentation) and “distance” (enacting segmentation but preferring integration). Data from 1,229 managers working in public and private organizations in Sweden was analyzed using polynomial regression analysis with response surface modeling and moderation analysis in SPSS Process. Findings showed that “integration congruence” was related with higher work-life conflict than “segmentation congruence.” Moreover, a U-shaped relationship between incongruence and work-life conflict was found: the more incongruence, the more work-life conflict. Specifically, “intrusion” was related to higher work-life conflict than “distance.” Finally, boundary control mitigated the effect of incongruence (especially “intrusion”) on work-life conflict. From our findings, we may conclude that work-life conflict is impacted differently depending on the type and level of boundary (in)congruence. Particularly enacted and/or preferred integration may be problematic when it comes to work-life conflict, rather than just (in)congruence *per se*. Moreover, boundary control can be viewed as a key factor in combating work-life conflict, especially among individuals who enact integration, but prefer segmentation. Taken together, our study contributes new and substantial knowledge by showing the importance for research and HRM-policies that take into account different types and levels of boundary (in)congruence, as these are associated with different levels of work-life conflict, which, in turn, are moderated by boundary control.

## Introduction

In 2020, everyday life changed dramatically for employees worldwide, resulting from the outbreak of the Covid-19 pandemic. To mitigate the spread of the contagion, large numbers of employees, more or less over a night, made a mandatory transition to home-based telework, i.e., carrying on one’s usual work-related duties from home through the use of information- and communication technologies (ICT) (Sullivan, 2003). Recent estimates pointed out that 558 million employees globally worked from home during the second quarter of 2020, accounting for 17.4% of the world’s workforce (Soares et al., 2021). During Spring 2020, as a result of the Swedish Public Health Agency’s recommendations on homeworking to reduce the spread of Covid-19, the number of employees working from home increased by 400% (Statistics Sweden, 2021a). Today, about 20% of all employees in Sweden work entirely from home, a ten-fold increase compared to before the pandemic. Only half of those had earlier experience with home working (The Swedish Internet Foundation, 2020). Moreover, a total of 42% of all employees now work from home at least part of their working hours (Statistics Sweden, 2021b), a number which is likely to increase in the wake of the pandemic (International Labour Organization [ILO], 2020; The Swedish Internet Foundation, 2020).

The developments in home-based telework clearly mark a fundamental shift regarding individuals’ work-nonwork boundaries, carrying both opportunities and challenges (Wajcman et al., 2008; Peters et al., 2009; Allvin et al., 2013). On the one hand, home-based telework empowers workers by enhancing their autonomy to organize their work as to accommodate the demands of work and nonwork in accordance with their own needs and preferences. On the other hand, however, self-organizing may also put increased demands on managing the increasingly blurred boundaries between work and nonwork. Indeed, in connection to the ongoing Covid-19 pandemic where many employees globally started working from home for the first time (Kramer and Kramer, 2020), a loss of control over work-nonwork boundaries has been frequently reported (Fisher et al., 2020).

Studies on the interplay between work and nonwork commonly focus on whether individuals experience a balance between multiple roles, be it work-nonwork enrichment or conflict (Bellavia and Frone, 2005; Greenhaus and Powell, 2006). The latter refers to a form of inter-role conflict (Frone et al., 1997a) that occurs when work and nonwork demands are mutually incompatible (Greenhaus and Beutell, 1985; Geurts et al., 2005), hindering individuals’ work and nonwork role enactment and performance (Frone et al., 1997b; Michel et al., 2011), either due to a lack of time or to strain built up in the work domain spilling over into the nonwork domain (Geurts et al., 2005). This has important implications given that work-life conflict can have serious consequences for stress-related ill-health, particularly in the Scandinavian countries (Finland, Norway, and Sweden) (Borgmann et al., 2019). Ill-health, in turn, has been associated with subsequent productivity loss and societal costs due to increased sickness absenteeism (Schmidt et al., 2019). For example, between 2014 and 2019, levels of long-term sickness absenteeism among managers in Sweden were shown to have sharply increased due to stress-related psychological ill-health, resulting from increased work demands and a loss of control over work-nonwork boundaries (Previa, 2019).

The boundary management literature (Nippert-Eng, 1996; Ashforth et al., 2000; Clark, 2000) offers a fruitful perspective to explain work-life conflict. In this perspective, boundary management strategies are presented along a continuum ranging from high on segmentation (degree of separating work and nonwork) to high on integration (degree of blending work and nonwork). Moreover, it is acknowledged that, due to contextual factors (such as the current Covid-19 pandemic), individuals’ actual behavior regarding the degree to which they segment or integrate work-nonwork, i.e., their enacted boundaries, are not always in line with their preferred boundaries, something which can have implications for their work-life conflict (Vaziri et al., 2020). In this context, work-life conflict is related to the degree of (in)congruence, or (mis)fit, between one’s enacted and preferred boundaries (Ashforth et al., 2000; Kreiner, 2006; Chen et al., 2009; Ammons, 2013). Several types of (in)congruence can be distinguished: “segmentation congruence” (enacting and preferring a high level of segmentation); “integration congruence” (enacting and preferring a high level of integration); as well as “intrusion” (incongruence in terms of enacting more integration than preferred) and “distance” (incongruence in terms of enacting more segmentation than preferred).

The assumed relationship between (in)congruence and work-life conflict (Kreiner et al., 2009) suggests that congruence between one’s enacted and preferred boundaries can reduce work-life conflict, whereas incongruence can increase work-life conflict. In view of this, it has been argued that it is important for individuals to be able to self-manage or self-control the transitions between their work and nonwork domains. This points to the importance of boundary control, defined as individuals’ perceptions of control over their work-nonwork boundary transitions (Kossek et al., 2012). Having a high degree of boundary control is believed to have the potential to reduce work-life conflict (Kossek et al., 2006, 2012).

However, it is not clear from the literature whether both types of congruence, that is, “segmentation congruence” and “integration congruence,” have similar effects on work-life conflict. In other words, does it matter for work-life conflict whether congruence relates to segmentation or integration? Moreover, it is not clear whether both types of incongruence, that is, “intrusion” versus “distance,” are equally detrimental for individuals’ work-life conflict. Also, it is not clear whether or how a particular degree as well as type of incongruence relates to the degree of self-control individuals perceive to have over their work-nonwork boundaries. Perhaps the impact of incongruence on work-life conflict may be less detrimental when individuals perceive that they can control the timing and frequency of their work-nonwork boundary transitions (Kossek et al., 2012). For instance, being able to self-determine whether one is available for work outside formal working hours may mitigate the effect of incongruence on work-life conflict, hence representing a form of psychological empowerment (Spreitzer, 1995) in terms of autonomously motivated integration behavior which may meet an individual’s basic psychological need for autonomy (Peters and Blomme, 2019).

This study, the first to the best of our knowledge, aims to contribute to the boundary management and work-life conflict literature by investigating the extent to which different types and levels of work-nonwork boundary (in)congruence relate to work-life conflict, and the extent to which boundary control moderates these relationships. Specifically, we examine whether (1) individuals’ segmentation and integration congruence, respectively; and (2) incongruence in terms of “intrusion” and “distance,” respectively, differently impact their experience of work-life conflict. In addition, we examine whether (3) boundary control can mitigate the effects of incongruence in terms of “intrusion” and “distance,” respectively, on work-life conflict.

### Enacted and Preferred Boundary Management

According to boundary theory, individuals’ enacted and preferred boundary management strategies can be presented along a segmentation-integration continuum (Nippert-Eng, 1996; Ashforth et al., 2000). At one end of the continuum lies high segmentation, which characterizes individuals who enact and prefer, respectively, relatively strong, or impermeable, work-nonwork boundaries (Ashforth et al., 2000; Hecht and Allen, 2009). At the other end of the continuum lies high integration, which characterizes individuals who enact and prefer, respectively, relatively weak, or permeable, work-nonwork boundaries (Ashforth et al., 2000; Hecht and Allen, 2009). As such, segmentation refers to keeping various aspects of the work and nonwork domains separated from one another, whereas integration refers to the degree to which various aspects of work and nonwork are merged or blended (cognitively, behaviorally, and/or physically) (Nippert-Eng, 1996; Ashforth et al., 2000; Kreiner, 2006). In this context, individuals’ role identity salience in terms of the importance they give to each of their multiple life roles (Thoits, 1992), such as work and nonwork, is a motivating factor in the enactment of their preferred boundaries. Individuals who for instance have a highly salient non-work role are motivated to protect the nonwork domain from work-related permeations, i.e., “protection effect” (Capitano et al., 2017). Therefore, they create less permeable boundaries around the nonwork domain. In other words, they enact segmentation. In contrast, individuals who have a highly salient work role are motivated to enact that role in the nonwork domain, and therefore, create permeable boundaries around the nonwork domain, i.e., “enactment effect” (Capitano et al., 2017). As such, they allow work-related permeations into the nonwork domain, thus enacting integration. Related to this, a recent study showed that the strongest motivating factor for enacting integration was an individual’s preference to integrate (Palm et al., 2020).

Both segmentation and integration are acknowledged to bring about costs and benefits. For example, segmentation can be beneficial when it comes to fulfilling work and nonwork roles (Dumas and Sanchez-Burks, 2015) and reducing work-life conflict (Powell and Greenhouse, 2010). However, in some cases, segmentation can lead to more work-life conflict, since integration, although more difficult, sometimes may be necessary in order to combine work and nonwork activities (Ashforth et al., 2000). More often, however, integration has been shown to be problematic, leading to, for example longer weekly work hours, poorer work-life balance (Mellner et al., 2014), more cross-role interruptions (Ashforth et al., 2000), more work-family conflict (Kossek et al., 2006; Matthews et al., 2014), and greater inter-role conflict (Bulger et al., 2007; Hecht and Allen, 2009). Hence, it can be argued that integration can create role blurring and work-nonwork conflict as individuals might find it more difficult to decide which role to pay attention to at a particular moment, which can create negative work-nonwork spillover (Ashforth et al., 2000).

### Boundary (in)Congruence

Boundaries can be regarded as social constructions that are shaped both by individuals’ desires and preferences and by cultural and institutional norms and practices (Moen and Chermack, 2005). As such, boundaries may or may not be consciously created by individuals, where structural conditions and norms in both the work and nonwork domains influence their enacted and preferred boundaries by offering possibilities, resources, constraints and/or demands which can either enhance or exacerbate perceptions of alignment, or boundary fit (Ammons, 2013). Thus, the boundary fit approach examines individuals’ enacted and preferred boundaries, and subsequent perceptions of fit, as shaped by and within the overall environmental context (Ammons, 2013). The only (to the best of our knowledge) study adopting a boundary fit approach (Ammons, 2013) showed that men and parents of young children had better boundary fit than women and those without caregiving responsibilities.

Another approach that has been more widely used in boundary management research is the person-environment P-E (mis)fit perspective (Kulka, 1979; Kreiner, 2006). This perspective focuses on the interaction between the individual and the environment and how this affects outcomes at the individual level. P-E (mis)fit has been defined as the congruence that occurs when employees and organizations are well matched (Kristof-Brown and Guay, 2011). It is assumed that when environmental conditions, such as workplace practices and norms, align with an individual’s boundary preferences, this results in boundary congruence which is associated with lower work-life conflict (Kreiner, 2006; Derks et al., 2016). However, when environmental conditions do not align with individuals’ boundary preferences, this results in boundary incongruence, which exacerbates work-life conflict (Ashforth et al., 2000; Kreiner, 2006; Chen et al., 2009; Kreiner et al., 2009). As such, various external (in)congruence sources (e.g., family members, superiors, subordinates, clients, and occupation) can either support or hinder individuals in enacting their preferred boundaries (Kreiner et al., 2009).

The present study looked into boundary (mis)fit, or (in)congruence, in terms of individuals’ enacted and preferred boundaries which is in line with the boundary fit approach. As such, we did not take into account external (in)congruence sources (Kreiner et al., 2009) which would be in line with the P-E (mis)fit perspective. However, it can be argued that, as individuals’ enacted and preferred boundaries are shaped by and within the environmental context (Ammons, 2013), in particular the ways in which boundaries are enacted can be regarded as a reflection of the possibilities, resources, constraints, and/or demands of the environment. Of course, there can be individual variations to some extent in how these external conditions are perceived and interpreted. This is supported by a recent study showing that the relationship between individuals’ preference for permeable boundaries and their permeability behavior was attenuated by pressure from one’s manager to prioritize work over nonwork (Capitano and Greenhaus, 2018). Therefore, we built our hypotheses, presented below, based on the concept of boundary (in)congruence (Kreiner et al., 2009) along Nippert-Eng’s (1996) segmentation-integration continuum.

From the examples of (external) boundary (in)congruence as related to work-life conflict, it can, on the one hand, be expected that work-life conflict would be lower when there is congruence between individuals’ enacted and preferred boundaries, irrespective of whether they enact and prefer segmentation or integration, as this reflects that the individual acts in line with his or her preferences (Kreiner, 2006). This would imply a non-linear relationship between boundary congruence and work-life conflict, such that work-life conflict would be expected to be lower when more congruence between individuals’ enacted and preferred boundaries occurs. More specifically, both when individuals enact and prefer segmentation and when they enact and prefer integration, respectively, work-life conflict can be expected to be lower.

On the other hand, although work-nonwork boundary congruence *per se* can be expected to be associated with lower work-life conflict, it could be argued that also the type of congruence may affect the experiencing of work-life conflict. Hence, when there is “segmentation congruence,” i.e., enacting and preferring a high level of segmentation, the impact of this type of congruence on work-life conflict may differ from that of “integration congruence,” i.e., enacting and preferring a high level of integration. Given that a large body of research has shown that both enacted and preferred integration can each have negative repercussions on various work-life outcomes (Ashforth et al., 2000; Bulger et al., 2007; Hecht and Allen, 2009; Mellner, 2016), including work-life conflict (Kossek et al., 2006; Matthews et al., 2014; Peters and Van der Heijden, 2019) and work-life balance (Mellner et al., 2014), it can be expected that individuals experience more work-life conflict when there is “integration congruence” in comparison with when there is “segmentation congruence.” As such, when individuals enact integration by opting for a high degree of work-nonwork transitions to take place, it can be expected to be associated with higher levels of work-life conflict, as these transitions may create role blurring and individuals may find it more difficult to decide which role to pay attention to at a particular moment. Hence, enacting a high level of integration, even when it is the preferred strategy, can make it more difficult for individuals to prevent negative spillover between the work and nonwork domains. Consequently, this can lead to higher work-life conflict as compared to individuals who both enact and prefer a high level of segmentation. By considering boundary congruence, in line with Nippert-Eng’s (1996) continuum, we can position segmentation on the higher end and integration on the lower end of this continuum and propose the following “differentiated boundary congruence hypothesis”:

**Hypothesis 1:** *There will be a negative linear relationship between boundary congruence and work-life conflict, such that work-life conflict will be higher when there is “integration congruence,” i.e., a high level of enacted and preferred integration, as compared to when there is “segmentation congruence,” i.e., a high level of enacted and preferred segmentation.*

Moreover, individuals do not always enact their preferred boundaries, leading to boundary incongruence which reflects boundary violations in the form of either “intrusion” or “distance” (Kreiner et al., 2009). This raises the question of whether these different types of incongruence associate differently with work-life conflict. However, in line with the P-E (mis)fit perspective, it could be expected that both types of incongruence can be associated with relatively high levels of work-life conflict; when individuals do not act in line with their preferred boundaries, frustration may build up, which can lead to strain that can impact work-life conflict (Kreiner et al., 2009). This would indicate a U-shaped relationship between boundary incongruence and work-life conflict. In other words, the higher the degree of incongruence between the enacted and preferred boundaries, the more strain individuals can be expected to experience, which in turn could be associated with higher levels of work-life conflict. We thus propose the following “boundary incongruence hypothesis”:

**Hypothesis 2:** *There will be a U-shaped relationship between boundary incongruence and work-life conflict, such that work-life conflict will be higher both when individuals experience “intrusion,” i.e., enacting more integration than preferred, and when individuals experience “distance,” i.e., enacting more segmentation than preferred.*

### The Moderating Role of Boundary Control

Boundary control represents individuals’ psychological interpretations of their control over their boundary environment (Kossek et al., 2012). The concept of boundary control can be related to Karasek’s (1979) job-strain model which posits that stress is prevalent when job demands exceed the degree of decision latitude needed by the individual to control these demands. Individuals with more boundary control are characterized by believing that they can control the timing, frequency, and direction of work-nonwork transitions to fit their preferences (Kossek et al., 2012). As such, boundary control can be regarded as individuals’ perceived ability to manage the boundaries between their work and nonwork domains.

Previous findings have shown that boundary control has the potential to reduce work-life conflict (Chen et al., 2009; Kossek et al., 2012) and may even be more important than individuals’ enacted and preferred boundaries, respectively, in relation to various work-life outcomes, such as work-life conflict. For instance, in an early study among teleworkers, Kossek et al. (2006) showed that the degree of boundary control was a stronger predictor of work-family conflict than work-nonwork integration. Another study among employees at a Swedish telecom company (Mellner et al., 2014) found that both a high preference for segmentation and high boundary control were each related to better work-life balance. This was particularly the case when a high preference for segmentation was combined with high boundary control. Moreover, Mellner (2016) showed that both high after-hours availability expectations, as a source of external (in)congruence (Kreiner et al., 2009), and enacted integration, in the form of work-related smartphone use during nonworking hours, were related to difficulties in letting go of work-related thoughts and feelings during leisure time, i.e., psychological detachment (Sonnentag and Fritz, 2007). Boundary control, however, was found to mitigate the effects of both after-hours availability expectations and work-related smartphone use during nonworking hours on psychological detachment (Mellner, 2016).

Currently, however, there is a lack of studies that simultaneously take into account boundary (in)congruence and boundary control. Thus, although previous studies have shown that enacted and preferred boundaries can each be associated with boundary control, and that all three concepts can be associated with work-life conflict, it remains unclear whether and how boundary control interacts with the effects of boundary (in)congruence on work-life conflict. Based on our second hypothesis, boundary incongruence will be detrimental to work-life conflict, and earlier findings regarding the importance of boundary control in relation to both enacted and preferred boundaries for various work-life outcomes, boundary control can be expected to moderate the relationship between boundary incongruence and work-life conflict. Moreover, given that previous findings have shown that integration can have negative repercussions of various work-life outcomes (Ashforth et al., 2000; Bulger et al., 2007; Hecht and Allen, 2009; Mellner et al., 2014), including work-family conflict (Kossek et al., 2006; Matthews et al., 2014; Peters and Van der Heijden, 2019), and that boundary control is an important factor in relation to various work-life outcomes (Mellner et al., 2014; Mellner, 2016), including work-life conflict (Kossek et al., 2006; Peters and Blomme, 2019), boundary control would be expected to mitigate the positive effect of boundary incongruence on work-life conflict especially in the case of “intrusion,” i.e., enacting more integration than preferred. For instance, obliging others’ (e.g., supervisors, colleagues, clients) actual and/or perceived expectations on being available for work outside of regular work hours when it is not in line with one’s preferences for keeping work and nonwork separated, may have less of a detrimental impact on the experience of work-life conflict when it is accompanied by the perception that one can control the timing and frequency of these work-nonwork transitions (Kossek et al., 2012). This kind of situation could be argued to represent a form of psychological empowerment (Spreitzer, 1995) in terms of autonomously motivated integration (Peters and Blomme, 2019). In other words, when individuals feel that they are in control of their work-nonwork boundaries, and thus choose to conform to conditions in their environment by enacting integration even when it does not match their general preference for segmentation, less strain arises that could spill over into the nonwork domain, which in turn can be associated with lower work-life conflict. Based on this, we propose the following “boundary control moderation hypothesis”:

**Hypothesis 3:** *Boundary control will moderate the presumed positive effect of boundary incongruence on work-life conflict, such that when incongruence is accompanied by higher levels of boundary control, work-life conflict will be lower. In particular, the moderating role of boundary control is expected to be especially pronounced in cases of incongruence in terms of “intrusion,” i.e., enacting more integration than preferred.*

## Materials and Methods

### Sample and Procedure

A web-survey was anonymously responded to by 1,599 (60% study sample response rate) managers working in the public and private sectors in Sweden. The participants were recruited to the study through their union membership. In Sweden, a majority (72%) of all professionals, including managers, are unionized (Kjellberg, 2019). The respondents in the present study belonged to three different unions: (1) representing occupations within health and welfare (study sample *n* = 605); (2) representing occupations within civil servant organizations (study sample *n* = 172); and (3) representing a large variety of different occupations and organizations within the private sector (study sample *n* = 822). In a first step, the respondents were informed about the survey through their respective union member magazines where the first author of the present study was interviewed about the survey. Next, an email was sent through the participating unions’ internal member email systems to all members who held a managerial position. This email included information about the survey, that the member registry unit at the respective unions would conduct a random selection of managerial members to participate in the survey, and that the members therefore might receive another e-mail from their respective unions with an invitation to participate in the study. This second e-mail, sent to the randomly selected members, included information from the first author of the present study on that participation was voluntary and that the respondents could decide to withdraw their participation at any given moment without explanation, and furthermore, that their participation was anonymous and that the data would be treated confidentially in accordance with the Swedish law on public access to information and secrecy (The Swedish Government, 2009: 400). In this e-mail, there was also a link to the web-survey. This link was independent of the participating unions’ network systems, but administered by a company that was also regulated by the Swedish law on public access to information and secrecy. This company provided an electronic web-survey tool. Finally, two reminders were sent via e-mail to all respondents, one after two weeks and one after one month. When the web-survey closed, all respondents’ e-mail addresses were erased automatically by the electronic web-tool system, and the questionnaire responses were de-identified and replaced by a code. Hence, there was complete respondent anonymity regarding the participating unions, the web-survey company, as well as the authors of the present study. The participating managers represented different organizational levels (CEO’s: *n* = 200; middle managers: *n* = 1,288; and expert managers such as manager of finance or personnel: *n* = 111). The analyses presented in this study included 1,229 respondents with complete data on all the study variables. In the study sample (see Table 1), 46% were male, and 39% was between 45 and 50 years old. Approximately 83% were cohabiting, 59% had children living in the household, and 96% worked full time. The questionnaire was in Swedish and all items for the measures used were translated from English by the first author of the present study and later back-translated into Swedish for accuracy verification by a native English- speaking professional translator.

**TABLE 1:** Bivariate correlations, means, standard deviations, percentages, and alpha reliabilities for all study variables.

	1	2	3	4	5	6	7	8
1. WLC	1							
2. EBM	−0.50**	1						
3. PBM	0.17**	0.33**	1					
4. BC	−0.55**	0.48**	−0.06*	1				
5. Age (45–54)	−0.18**	0.13**	–0.03	0.09**	1			
6. Male	–0.04	−0.12**	−0.17**	–0.04	0.01	1		
7. Single	–0.02	0.02	–0.01	0	–0.01	–0.08*	1	
8. Children	−0.06*	−0.10**	0.01	−0.06*	–0.31**	0.02	0.12**	1
M	1.67	4.36	5.39	3.72	–	–	–	–
S.D.	0.60	1.45	1.54	0.98	–	–	–	–
Per cent	–	–	–	–	38.80%	46%	83%	60%
Alpha	0.91	0.84	0.9	0.91	–	–	–	–

WLC = Work-Life Conflict; EBM = Enacted Boundary-Management (high values = enacted segmentation); PBM = Preferred Boundary-Management (high values = preferred segmentation); BC = Boundary Control. *p < 0.05; **p < 0.01 (2-tailed).

### Measures

#### Work-Life Conflict

The SWING scale (Geurts et al., 2005) was used to measure work-life conflict (9 items). Example item: “How often does it happen that your work obligations make it difficult for you to feel relaxed at home?.” A 4-point Likert-type scale (1 = almost never; 4 = almost always) was used. The item response values were summated to create a scale where higher scores corresponded to higher levels of work-life conflict (Cronbach’s α = 0.91).

#### Enacted Boundary Management

The first five items from Kossek et al. (2012) work-life indicator scale were used to capture enacted boundary management (EBM), measured on a 5-point Likert-type scale (1 = strongly disagree; 5 = strongly agree). Example item: “I respond to work-related communications (e.g., emails, texts, and phone calls) during my personal time away from work.” The item response values were reversed and summated to create a scale where higher scores corresponded to higher levels of enacted boundary management in terms of high enacted work-nonwork segmentation (Cronbach’s α = 0.84).

#### Preferred Boundary Management

To measure preferred boundary management (PBM), Kreiner’s (2006) four-item scale for capturing desire for segmentation was used, measured on a 7-point Likert-type scale (1 = strongly disagree; 7 = strongly agree). Example item: “I prefer to keep work at the workplace.” The item response values were summated to create a scale where higher scores corresponded to higher levels of preferred boundary management in terms of high preference for segmentation (Cronbach’s α = 0.90).

#### Boundary Control

The three boundary control items from Kossek et al. (2012) work-life indicator scale were utilized to capture this variable, measured on a 5-point Likert-type scale (1 = strongly disagree; 5 = strongly agree). Example items: “I control whether I am able to keep my work and personal life separate” and “I control whether I combine my work and personal life activities throughout the day.” The item response values were summated to create a scale where higher scores corresponded to higher levels of boundary control (Cronbach’s α = 0.91).

#### Control Variables

We controlled for four variables: age (1 = under 35, 2 = 35-44, 3 = 45-54, 4 = older than 55), gender (female = 0, male = 1, other = 3, 4 = don’t want to answer, where categories 3 and 4 were treated as missing data), marital status (0 = married or cohabiting, 1 = single, 3 = don’t want to answer, where category 3 was treated as missing data), and children in the household (0 = no, 1 = yes, 3 = don’t want to answer, where category 3 was treated as missing data), in our analyses, as these variables could be expected to impact work-life conflict as well (Remery and Schippers, 2019).

### Analyses

To reduce potential common method bias effects, we conducted several *a priori* analyses. We used a two-step approach, following Podsakoff and Organ (1986), in which we first conducted a one-factor test in which all items measuring the principal constructs were entered into a principal component factor analysis, using the extraction method without rotation for one fixed factor (SPSS version 24 for Windows). The results showed that one factor explained less than 50% of the variance (36.85%), providing an initial indication of no common method variance (CMV) (Harman, 1976). Second, we tested the correlation between the constructs, which should be less than 0.9 (Bagozzi et al., 1991). Table 1 shows that the highest correlation between any two constructs was –0.55 (between work-life conflict and boundary control). Hence, no indication of CMV was found in the data.

To test the (in)congruence hypotheses, polynomial regression analysis and response surface modeling was used (Edwards and Parry, 1993). The scales were rescaled to use the same scale for both enacted and preferred boundary management, and the scales were also centered to reduce multi-collinearity between the component measures, that is, enacted and preferred boundary management, and their associated higher order terms (Aiken and West, 1991).

To test the boundary control moderation hypothesis, we applied the block variable approach suggested by Cable and Edwards (2004). This involves obtaining a single coefficient that summarizes the effects of a set of conceptually related variables (ibid.). In this study, to test the U-shaped relationship between incongruence based on the preferred (X-variable) and enacted (Y-variable) boundaries and their product term (X^∗^Y) and squared values (X^2^ and Y^2^), we constructed a block variable by first regressing the dependent variable, work-life conflict, on the five polynomial terms (X, Y, X^∗^Y, X^2^ and Y^2^) presented above. We then used the respective weights, which were the estimated regression coefficients in the polynomial regression (*b*_1_X + *b*_2_Y + *b*_3_X^2^ + *b*_4_XY + *b*_5_Y^2^) and combined the five terms into a block variable as a weighted composite that summarized the effects of enacted/preferred boundary incongruence on work-life conflict (Edwards and Cable, 2009).

To assess the joint impact of enacted and preferred boundaries on work-life conflict, it is important to take levels of enacted and preferred boundaries into account. Similarity patterns are used to assess different types of similarities between pairs of predictor variables. Such patterns are based on two main assumptions: that there is an optimal match between two variables (such as enacted and preferred boundaries), and that deviation from this optimal match leads to less optimal outcomes, with bigger deviations having more impact on the outcomes. Therefore, by utilizing similarity patterns, it can be estimated whether there is an optimal level of similarity between enacted and preferred boundaries when predicting work-life conflict. Polynomial regression analysis can be used to investigate the linear effects of predictor variables, the quadratic effects of predictor variables, and the effects of the interaction between the predictor variables. Specifically, an intercept (b0), a linear (b1), and quadratic (b3) effect of enacted boundaries, a linear (b2) and quadratic (b5) effect of preferred boundaries, and an interaction between the linear effects of enacted and preferred boundaries (b4) can be estimated.

Due to the combination of quadratic terms and an interaction term, interpretations of polynomial regressions are notoriously difficult. To facilitate interpretation, response surface analysis has been developed (Edwards and Parry, 1993; Rhoades Shanock et al., 2010). Response surface analysis provides a visual representation of the outcomes of polynomial regressions based on similarity and dissimilarity between two variables. In the present study, the x-axis indicates the level of enacted boundaries, the y-axis indicates the level of preferred boundaries, and the z-axis indicates the level of work-life conflict.

Two parameters (*a*1 and *a*2) represent effects along a line of congruence (similarity). In our study, the line of congruence is the line where enacted boundaries and preferred boundaries have similar scores. They indicate a linear slope (*a*1) and a quadratic slope (*a*2) for the effect of congruence between enacted and preferred boundaries on work-life conflict. Thus, a finding of significant effects would indicate that congruence impacts work-life conflict. Other linear (*a*3) and quadratic (*a*4) terms indicate whether there is a dissimilarity effect of enacted and preferred boundaries on work-life conflict, along a line of incongruence. The linear slope effect (*a*3) indicates the likelihood for higher work-life conflict when the enacted boundaries are higher than the preferred boundaries on work-life conflict. The quadratic effect (*a*4) indicates whether work-life conflict is especially likely at high or low levels of dissimilarity. Thus, a finding of significant effects would indicate that incongruence impacts work-life conflict.

Lastly, we conducted moderation analysis using the PROCESS macro (Hayes, 2013) in SPSS, with the block variable “boundary incongruence” as the independent variable, boundary control as the moderating variable, and work-life conflict as the dependent variable. We examined the conditional effects using bootstrap as a bias-correction percentile method with 10,000 samples (Cable and Edwards, 2004) and calculated bias-corrected confidence intervals (Edwards, 2002). The proposed moderation is supported if the confidence interval of the indirect effect does not include zero.

## Results

### Preliminary Analyses

In Table 1, the correlations and descriptive statistics of the study variables are presented.

### Hypothesis Testing

**FIGURE 1:**
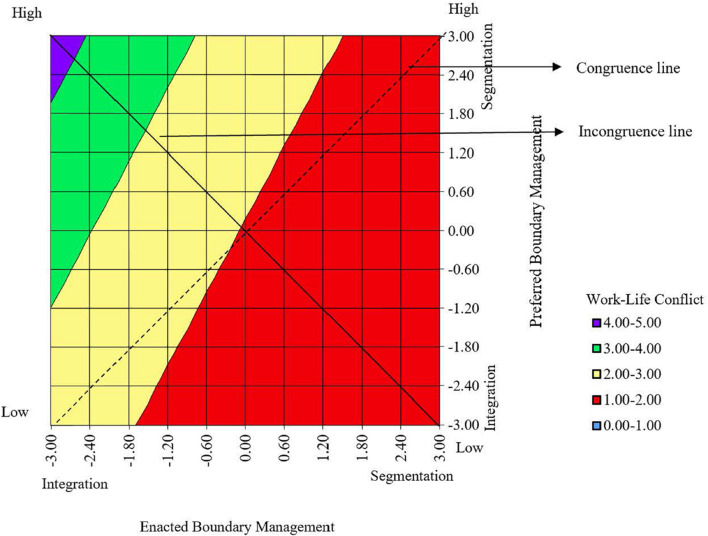
Graphical aid for interpreting congruence and incongruence lines in the response surface.

Table 2 shows the results of the polynomial regression analysis. Hypothesis 1, the differentiated boundary congruence hypothesis, predicted a negative, linear relationship between boundary congruence and work-life conflict, where levels of congruence were positioned on a continuum from “segmentation congruence” (high) to “integration congruence” (low). The congruence line of the response surface for enacted and preferred boundaries had a significant negative linear slope (*a*1) (*b* = –0.10, *p*<.01). This indicates that work-life conflict was higher for “integration congruence,” and lower for “segmentation congruence,” thus supporting Hypothesis 1. In Figure 1, this is visualized by the dashed line running from the bottom left-hand corner, where both enacted and preferred boundary management is low (i.e., “integration congruence”), to the top right-hand corner, where both enacted and preferred boundary management is high, (i.e., “segmentation congruence”). It shows that the “integration congruence” is situated in the area where work-life conflict is higher (ranging from 2 to 3) whereas the “segmentation congruence” is situated in the area where work-life conflict is lower (ranging from 1 to 2). Hence, even though all points along the congruence line represent boundary congruence, “integration congruence” was found to be associated with higher levels of work-life conflict than was “segmentation congruence.”

**TABLE 2:** Polynomial regression analysis results of Preferred and Enacted Boundary Management, respectively, and their interaction, i.e., Boundary Management (in)Congruence, predicting Work-Life Conflict.

Outcome variable	WLC
Constant	1.759 (30.51)*
Age (45–54)	–0.09 (–3.95)*
Male	–0.06 (–2.77)*
Single	–0.01 (–0.48)
Children	–0.04 (–1.64)
EBM Centered (x)	–0.51 (–14.23)*
PBM Centered (y)	0.28 (8.92)*
EBM Squared	0.16 (6.24)*
Enacted (x)*Preferred (y) Boundary Management	–0.21 (–5.79)*
PBM Squared	0.05 (1.80)
F	97.17
*Df*	3–1220
*R* ^2^	0.42
**Congruence (enacted-preferred congruence line)**	
Slope (*a*1)	–0.10 (0.02)*
Curvature (*a*2)	0.01 (0.01)
**Incongruence (enacted-preferred incongruence line)**	
Slope (*a*3)	–0.33 (0.02)*
Curvature (*a*4)	0.09 (0.01)*

WLC = Work-Life Conflict; EBM = Enacted Boundary Management (high values = enacted segmentation); PBM = Preferred Boundary Management (high values = preferred segmentation); BC = Boundary Control. N = 1,229. *p < 0.001; the values reported are standardized beta coefficients; standard errors are in the parentheses.

Hypothesis 2, the boundary incongruence hypothesis, predicted a U-shaped relationship between boundary incongruence and work-life conflict. To test Hypothesis 2, we used the computed block variable “boundary incongruence,” using the estimated coefficients to predict work-life conflict. As expected, the incongruence line of the response surface for enacted and preferred boundary management had a significant positive curvature (*a*4) (*b* = .09, *p* < .01) (see Table 3). This indicates that higher work-life conflict was associated with boundary incongruence toward both extremes of the boundary management incongruence continuum, that is, both when there was “intrusion” and when there was “distance,” thus lending support for Hypothesis 2. This is presented in Figure 1, where the continuous line of incongruence runs from the upper left-hand corner, where enacted boundary management is low (high level of enacted integration) and preferred boundary management is high (high level of preferred segmentation), that is, “intrusion,” to the lower right-hand corner, where enacted boundary management is high (high level of enacted segmentation) and preferred boundary management is low (high level of preferred integration), that is, “distance.” Moreover, it shows that the “intrusion” incongruence is situated in the area where work-life conflict is higher (ranging from 4 to 5), whereas the “distance” incongruence is situated in the area where work-life conflict is lower (ranging from 1 to 2).

**TABLE 3:** Moderation analysis results with the PROCESS macro of the block variable Boundary Management Incongruence predicting Work-Life Conflict, with Boundary Control as a moderator.

Outcome variable	WLC	
Predictor	*B*	*p*
Intercept	2.45 (0.08)	0
BMI	4.70 (0.50)	0
BC	–0.18 (0.02)	0
BMI*BC	–0.34 (0.15)	0.02
Age (45–54)	–0.06 (0.01)	0
Male	–0.08 (0.03)	0
Single	–0.03 (0.03)	0.43
Children	–0.04 (0.03)	0.1
Model *R*^2^	0.49	
*F*	166.1	0

*WLC = Work-Life Conflict; BMI = Boundary Management Incongruence (low = “distance,” i.e., enacting segmentation, but preferring integration; high = “intrusion,” i.e., enacting integration, but preferring segmentation); BC = Boundary Control.*

*N = 1,229. The coefficients reported are unstandardized; standard errors are in the parentheses.*

The above was further confirmed as, unexpectedly, the incongruence line of the response surface for enacted and preferred boundary management also had a significant negative slope (*a*3) (*b* = –0.33, *p* ≤ 0.01) (see Table 3). This implies a linear relationship: work-life conflict was higher for “intrusion” than for “distance.” Thus, the findings showed support not only for Hypothesis 2, which predicted that work-life conflict would be associated with boundary incongruence toward both extremes of the boundary incongruence continuum, but they also evidenced what can be labelled as an effect of *differentiated* boundary incongruence on work-life conflict.

**FIGURE 2:**
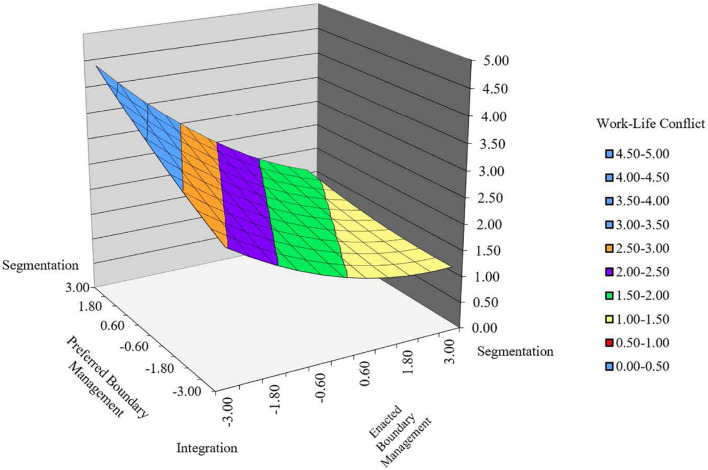
Response surface with associated lines of congruence and incongruence of the polynomial regressions for enacted boundary-management and preferred-management explaining work-life conflict.

Taken together, the overall patterns of boundary (in)congruence are shown in Figure 2, which presents the response surface of work-life conflict at different types and levels of boundary (in)congruence. As can be seen in the upper left-hand corner, the highest levels of work-life conflict (ranging from 3 to 5), were found in the case of incongruence in terms of “intrusion,” i.e., enacting more integration than preferred, followed by “integration congruence,” i.e., enacting and preferring a high level of integration (work-life conflict ranging from 2.5 to 3). Next, incongruence in terms of “distance,” i.e., enacting more segmentation than preferred, was associated with work-life conflict (ranging from 1.5 to 2). The lowest levels of work-life conflict (ranging from 1 to 1.5), as can be seen in the lower right-hand corner, were found in the case of “segmentation congruence,” i.e., enacting and preferring a high level of segmentation.

**FIGURE 3:**
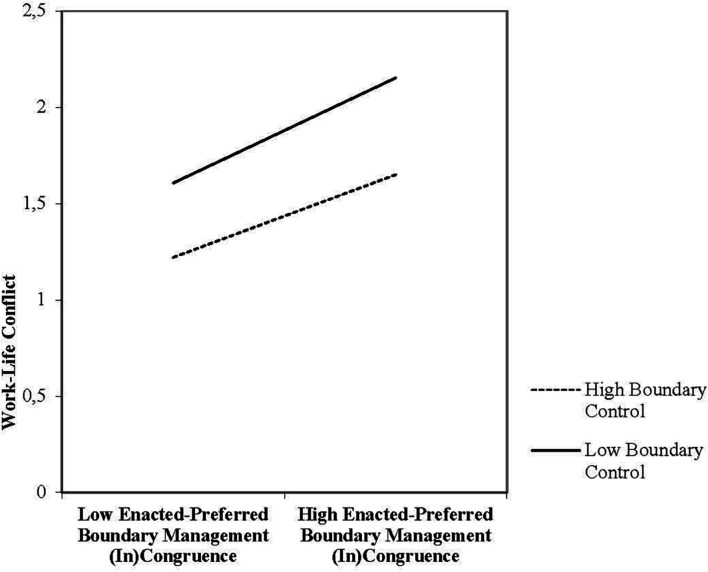
Moderation of the effect of Boundary Management Incongruence (on a continuum from low = “distance” to high = “intrusion”) Work-Life Conflict at values of the moderator Boundary Control.

Finally, Hypothesis 3 predicted that boundary control would moderate the effect of boundary incongruence on work-life conflict, especially in the case of “intrusion,” i.e., enacting more integration than preferred. To examine the moderating effect of boundary control on the relationship between incongruence and work-life conflict, we generated 95% bias-corrected confidence intervals (Preacher and Hayes, 2004) for the hypothesized conditional effects. As can be seen in Table 3, the direct effect of the block variable “boundary incongruence” on work-life conflict, before the inclusion of boundary control as moderator, was significant and positive (*b* = 4.70, *p* < 0.001). Furthermore, the effect of the interaction between boundary incongruence and boundary control on work-life conflict was also significant and negative (*b* = –0.34, *p* < 0.05), indicated by the confidence interval from the bootstrap analysis excluding zero [–0.6268, –0.0481]. These findings lend support to Hypothesis 3 as boundary control was shown to mitigate the effects of boundary incongruence on work-life conflict, especially when “intrusion” was accompanied by high levels of boundary control. See Figure 3.

## Discussion and Conclusion

The present study examined whether different types and levels of boundary (in)congruence impact differently on work-life conflict, and if different types and levels of boundary incongruence relate to perceived level of self-control with regard to managing work-nonwork boundaries.

Boundary congruence was associated with lower work-life conflict as compared to boundary incongruence. Although it was not specifically addressed in any of our hypotheses, this finding could be expected from a P-E (mis)fit perspective (Kristof-Brown and Guay, 2011), as has also been shown in previous studies (Kreiner, 2006; Derks et al., 2016).

However, as our aim was to move beyond the study of boundary (in)congruence *per se*, we instead focused on whether type and level of (in)congruence played a role in the degree of work-life conflict experienced.

Indeed, as expected from our first hypothesis, “integration congruence” was more positively associated with work-life conflict than “segmentation congruence.” Thus, even when there was boundary congruence, this finding reveals that the specific type of congruence matters, as “integration congruence” was shown to be positively associated with work-life conflict compared to “segmentation congruence.” As such, our findings further those of previous studies, which have shown that both enacted and preferred integration can be associated with negative effects on various work-life outcomes (Ashforth et al., 2000; Bulger et al., 2007; Hecht and Allen, 2009; Mellner et al., 2014; Mellner, 2016), including work-life conflict (Kossek et al., 2006; Matthews et al., 2014; Peters and Van der Heijden, 2019). This was done by showing that enacted integration can be problematic in terms of higher work-life conflict, even when this is in line with one’s preferred boundaries, that is, when there is “integration congruence.” One explanation that has been put forth regarding the impact of integration on work-life conflict is that integration may create role blurring and conflict between work and nonwork roles, as individuals might find it more difficult to decide which role they should pay attention to at a particular moment, which makes it difficult for them to prevent negative spillover from work into nonwork (Ashforth et al., 2000). Extending this explanation, based on our findings, it seems that the positive association between enacting a high level of integration and work-life conflict, i.e., higher work-life conflict, overrides the negative association between congruence and work-life conflict, i.e., lower work-life conflict, but only in the case of “integration congruence.” This was further underscored by the fact that “integration congruence” was associated with some of the highest levels of work-life conflict, whereas “segmentation congruence” was associated with the lowest levels of work-life conflict.

When it comes to boundary incongruence, we found a U-shaped relationship between boundary incongruence and work-life conflict. Work-life conflict was higher in relation to both “intrusion,” and “distance.” This was in line with our second hypothesis, based on the expectation that when individuals do not act in line with their preferred boundaries, this represents boundary violations that may cause the building up of frustration, which can lead to strain that can increase work-life conflict (Kreiner et al., 2009). Unexpectedly, however, the different types of incongruence were also found to associate differently with work-life conflict. Specifically, “distance” was shown to have a weaker association with work-life conflict than “intrusion.” Interestingly, “distance” was associated with the next lowest levels of work-life conflict, after “segmentation congruence.” As such, our findings go beyond those of earlier studies, which have shown that both enacted and preferred segmentation are each generally more beneficial when it comes to fulfilling work-nonwork roles (Dumas and Sanchez-Burks, 2015), being associated with lower work-life conflict (Powell and Greenhouse, 2010; Peters and Van der Heijden, 2019) and higher work-life balance (Mellner et al., 2014). More specifically, by taking enacted and preferred boundaries into account simultaneously, it was shown that enacting segmentation, even when doing so was incongruent with one’s preferred boundaries, was associated with lower work-life conflict. This suggests that the positive impact of incongruence on work-life conflict, i.e., higher work-life conflict, is counteracted, i.e., managers experienced less work-life conflict when enacting a higher level of segmentation although preferring more integration, i.e., “distance.” Hence, although one is not acting in line with one’s preferred boundaries, enacted segmentation appeared to be negatively associated with work-life conflict, i.e., lower work-life conflict, which overrides the positive association between incongruence and work-life conflict, i.e., higher work-life conflict. In contrast, enacting integration, especially when this was incongruent with one’s preferred boundaries, i.e., “intrusion,” was associated with the highest levels of work-life conflict. This may be explained by that the positive association between boundary incongruence and work-life conflict, i.e., higher work-life conflict, is exacerbated when enacting a higher level of integration although preferring more segmentation, i.e., “intrusion.” Thus, when not acting in line with one’s preferred boundaries, the detrimental impact of enacted integration on work-life conflict, i.e., higher work-life conflict, seems to make one more vulnerable to the positive impact of incongruence on work-life conflict, i.e., higher work-life conflict.

Finally, in line with our third hypothesis, we found that perceived boundary control mitigated the positive relationship between boundary incongruence and work-life conflict; when incongruence was accompanied by higher levels of boundary control, work-life conflict was lower. This was especially the case with regard to “intrusion” as compared to “distance” – a finding that was also in line with our expectations. Our findings extend those from earlier studies showing that boundary control is important for various work-life outcomes (Mellner et al., 2014; Mellner, 2016), including work-life conflict (Kossek et al., 2006; Peters and Blomme, 2019); low boundary control is associated with enacted integration (Leonardi et al., 2010; Dumas and Sanchez-Burks, 2015; Mellner, 2016); and high boundary control is associated with both enacted (Ashforth et al., 2000; Peters and Blomme, 2019); and preferred segmentation (Mellner et al., 2014). More specifically, although previous studies have shown that enacted and preferred boundaries can each be associated with boundary control, and that all three concepts can be associated with work-life conflict, we simultaneously took into account boundary incongruence and boundary control, by examining whether and how boundary control interacts with the effect of boundary incongruence on work-life conflict. In doing this, we showed that boundary control is an important factor that can reduce the strain associated with boundary incongruence, especially in the case of “intrusion.” One potential explanation for this particular kind of situation is that boundary control can reflect a form of psychological empowerment (Spreitzer, 1995) in terms of autonomously motivated integration (Peters and Blomme, 2019). In other words, when individuals feel that they are in control over the timing and frequency of work-nonwork transitions (Kossek et al., 2012), and thus choose to conform to conditions in their environment by enacting integration even when it does not match their general preference for segmentation, less strain arises that could spill over into the nonwork domain, which in turn can be associated with lower work-life conflict.

### Strengths, Limitations, and Future Research

The present study can be seen as a strong contribution to the literature on boundary management and work-conflict, as it is one of the first to investigate the interplay between individuals’ enacted and preferred boundaries through examining the effects of different types and levels of boundary (in)congruence on work-life conflict, and the moderating role of boundary control in these relationships. Moreover, our study included 1,229 managers within different occupations and organizations in both the private and public sector. As such, one of the limitations of much work-nonwork research where samples are drawn from one occupation or one organization reducing the generalizability of the findings (Kossek and Ozeki, 1998 in Kreiner, 2006) was overcome. In addition, the inclusion of several occupations and organizations lends the findings additional strength as (in)congruence needs to be investigated in various environments and settings (Ostroff and DuBois, 1993 in Kreiner, 2006).

Despite the contributions of our study, there are limitations that should be acknowledged. First, our study was based on cross-sectional, single source data. However, we employed procedural remedies (Podsakoff et al., 2003) to stave off potential problems that might arise from having a common rater. We also used measures from established questionnaires which were found to have good psychometric properties. Moreover, the items for different measures had different scale anchors. This reduces the risk of adopting a personal response style irrespective of the item content. Second, related to the use of cross-sectional data, longitudinal processes, including causal inferences, cannot be made. Moreover, we did not include measures of role salience identity, i.e., the importance, in terms of norms, values, beliefs, and goals, that individuals attach to their role identities (Thoits, 1992; Settles, 2004), where people may be either “work centric” or “nonwork centric.” However, it is reasonable to assume that, in modern working life, with its increasing numbers of dual-earner couples and where many men and women have multiple work and nonwork roles (Ferrarini and Duvander, 2010; Kossek et al., 2012), a large proportion of individuals will be “dual centric,” i.e., they identify strongly with both their work and nonwork roles. This can be expected to be the case in the present study as 96% of the participants were working full-time, 83% were cohabiting, and 59% had children living in the household.

Based on the findings of the present study, it would be of interest for future research to focus on identifying factors in the environmental context, such as (actual and/or perceived) possibilities, resources, constraints and/or demands in both work and nonwork, that may be related to different types and levels of boundary (in)congruence regarding individuals’ enacted and preferred work-nonwork boundaries. Also, since boundary control can be viewed as a key factor in the relationship between boundary incongruence and work-life conflict, future studies may be concerned with how perceptions of boundary control are associated with the interplay between different types and levels of incongruence and related identified factors in the environmental context.

Moreover, previous research on work-life balance, in terms of both enrichment and conflict (Bellavia and Frone, 2005; Greenhaus and Powell, 2006), has either investigated enrichment or conflict separately or focused on bidirectional associations and used cross-sectional data (LaPierre and McMullan, 2016; Allen et al., 2019). Therefore, it would be of interest for future studies to examine changes of individual profiles of work-life balance, both enrichment and conflict taken together, over time that account for intra-individual variability (Eby et al., 2016). This kind of research could answer questions concerning whether and how individuals transition between enrichment and conflict, and why some individuals experience lower enrichment and/or greater conflict while others experience increased enrichment and/or reduced conflict in relation to boundary (in)congruence, perceived boundary control, and the environmental context.

Finally, there is a current lack of understanding of the embeddedness of individuals’ response patterns regarding conditions of boundary (in)congruence, as well as of perceptions of boundary control, within the context of individual differences. Traditionally, work-nonwork researchers have treated individual differences as control variables rather than as aspects of work-nonwork processes that may be important in their own right (Eby et al., 2005). For instance, in the light of large numbers of employees worldwide having made a transition to mandatory home-based telework during the ongoing Covid-19 pandemic with related reports of loss of work-nonwork boundaries (Fisher et al., 2020), little attention has previously been paid to the association between the interplay between gender, marital status and parenthood taken together, and home-based telework (Asgari et al., 2014; Paleti, 2016). However, one recent study showed that the presence of children at home during voluntary home-based telework increased work-life conflict and aggravated gender differences (Zhang et al., 2020). Thus, one promising avenue for future research would be to utilize more comprehensive models that are able to take individual differences into account leading to findings that may be able to increase our understanding of the individual nature of work-nonwork boundary management processes.

### Conclusion and Implications

Several important conclusions can be drawn from of our findings. First, work-life conflict is impacted differently depending on the type and level of boundary (in)congruence, rather than just (in)congruence *per se*. Specifically, enacted and/or preferred segmentation can be regarded as beneficial in terms of reduced work-life conflict. In contrast, enacted and/or preferred integration can be regarded as problematic in terms of increased work-life conflict. Second, when individuals perceive that they have the agency needed to decide how to interact with their own work-nonwork boundaries, i.e., boundary control, the detrimental effects of particularly boundary incongruence in terms of “intrusion” on work-life conflict can be mitigated.

Our study has important implications for human resource practices within organizations, as todays’ working life is characterized by increased opportunities as well as challenges related to the self-organization of one’s work (Wajcman et al., 2008; Peters et al., 2009; Allvin et al., 2013), including demands on managing increasingly blurred work-nonwork boundaries. This is particularly the case given that telework, which during the current Covid-19 pandemic has been associated with reports of a loss of control over work-nonwork boundaries (Fisher et al., 2020), is expected to be here to stay and even increase (International Labour Organization [ILO], 2020). Our findings clearly indicate that, to combat work-life conflict, organizations would gain from supporting employees in creating and maintaining strong work-nonwork boundaries. This applies particularly to organizations characterized by a culture where employees are expected to be available on work-related issues during leisure time. Human resource policies may be developed that reflect legitimate segmentation norms (Kok et al., 2015). More specifically, policies need to include the right to undisturbed leisure time, keeping work within contractual work hours, and sufficient recovery time between work shifts. This would protect the work-nonwork boundaries of all employees regardless of boundary preferences, and subsequently, reduce work-life conflict.

## Data Availability Statement

The raw data supporting the conclusions of this article will be made available by the authors, without undue reservation.

## Ethics Statement

The studies involving human participants were reviewed and approved by the Regional Ethics Committee in Stockholm. Written informed consent for participation was not required for this study in accordance with the national legislation and the institutional requirements.

## Author Contributions

CM: conceptualization, methodology, investigation, writing – original draft, writing – review and editing, supervision, project administration, and funding acquisition. PP: conceptualization, methodology, investigation, writing – original draft, and writing – review and editing. MD: methodology, formal analysis, and writing – original draft (statistical analysis). ST: conceptualization and writing – review and editing. All authors contributed to the article and approved the submitted version.

## Conflict of Interest

The authors declare that the research was conducted in the absence of any commercial or financial relationships that could be construed as a potential conflict of interest.

## Publisher’s Note

All claims expressed in this article are solely those of the authors and do not necessarily represent those of their affiliated organizations, or those of the publisher, the editors and the reviewers. Any product that may be evaluated in this article, or claim that may be made by its manufacturer, is not guaranteed or endorsed by the publisher.
